# Temporal and nutritional effects on the weaner pig ileal microbiota

**DOI:** 10.1186/s42523-021-00119-y

**Published:** 2021-08-28

**Authors:** Jolinda Pollock, Laura Glendinning, Lesley A. Smith, Hamna Mohsin, David L. Gally, Michael R. Hutchings, Jos G. M. Houdijk

**Affiliations:** 1 Animal and Veterinary Sciences, Scotland’s Rural College (SRUC), Edinburgh, UK; 2 SRUC Veterinary Services, Scotland’s Rural College, Edinburgh, UK; 3 The Roslin Institute and Royal (Dick) School of Veterinary Studies, University of Edinburgh, Edinburgh, UK

**Keywords:** Microbiome, Microbiota, Ileum, Gut, Weaning, Pigs, Nutrition, Protein, Metagenomics

## Abstract

**Background:**

The porcine gastrointestinal microbiota has been linked to both host health and performance. Most pig gut microbiota studies target faecal material, which is not representative of microbiota dynamics in other discrete gut sections. The weaning transition period in pigs is a key development stage, with gastrointestinal problems being prominent after often sudden introduction to a solid diet. A better understanding of both temporal and nutritional effects on the small intestinal microbiota is required. Here, the development of the porcine ileal microbiota under differing levels of dietary protein was observed over the immediate post-weaning period.

**Results:**

Ileal digesta samples were obtained at post-mortem prior to weaning day (day − 1) for baseline measurements. The remaining pigs were introduced to either an 18% (low) or 23% (high) protein diet on weaning day (day 0) and further ileal digesta sampling was carried out at days 5, 9 and 13 post-weaning. We identified significant changes in microbiome structure (P = 0.01), a reduction in microbiome richness (P = 0.02) and changes in the abundance of specific bacterial taxa from baseline until 13 days post-weaning. The ileal microbiota became less stable after the introduction to a solid diet at weaning (P = 0.036), was highly variable between pigs and no relationship was observed between average daily weight gain and microbiota composition. The ileal microbiota was less stable in pigs fed the high protein diet (P = 0.05), with several pathogenic bacterial genera being significantly higher in abundance in this group. Samples from the low protein and high protein groups did not cluster separately by their CAZyme (carbohydrate-active enzyme) composition, but GH33 exosialidases were found to be significantly more abundant in the HP group (P = 0.006).

**Conclusions:**

The weaner pig ileal microbiota changed rapidly and was initially destabilised by the sudden introduction to feed. Nutritional composition influenced ileal microbiota development, with the high protein diet being associated with an increased abundance of significant porcine pathogens and the upregulation of GH33 exosialidases—which can influence host-microbe interactions and pathogenicity. These findings contribute to our understanding of a lesser studied gut compartment that is not only a key site of digestion, but also a target for the development of nutritional interventions to improve gut health and host growth performance during the critical weaning transition period.

**Supplementary Information:**

The online version contains supplementary material available at 10.1186/s42523-021-00119-y.

## Background

There have been many studies in recent years on the development of the porcine gut microbiota, which have primarily focussed on faecal microbial communities. Comparatively less studies have been done on the small intestinal microbiota, primarily due to the challenge of obtaining samples and the assumption that faecal material can be considered a proxy for upstream gut microbiota interactions [1]. From previous work, we know that the porcine microbiota composition varies between gut sections [1–4] and the impact of treatment effects have been investigated specifically on the ileal microbiota composition at single time points [1, 5–7]. However, there are few studies that explore temporal development and nutritional influences on the small intestinal microbiota.

The immediate post-weaning period in pigs is a critical development stage, where disease risk is increased due to sudden changes in diet, environment and sow removal. In previous work, we have shown that both enterotoxigenic *Escherichia coli* (an important causative agent of post-weaning diarrhoea) exposure and an increase in dietary crude protein (CP) level have a significant impact on ileal microbiota composition in weaner pigs, in the absence of changes at faecal level [1]. The manipulation of dietary CP levels has been considered as a control measure for post-weaning diarrhoea, as its reduction has been shown to lower disease severity [8, 9]. In our previous work [1], we considered the spatial variation in the gut microbiota in response to dietary CP manipulation and enterotoxigenic *Escherichia coli* exposure but have yet to explore temporal changes in ileal microbiota composition. Here, we compared ileal bacterial communities from pigs fed a low CP (LP) and a high CP (HP) diet to gain information on the effects of CP nutrition on ileal microbiota composition and function through the immediate post-weaning period, with consideration of how weight gain may be linked to ileal microbiota composition.

## Results

### Taxonomic description of the ileal microbiota

**Fig. 1:**
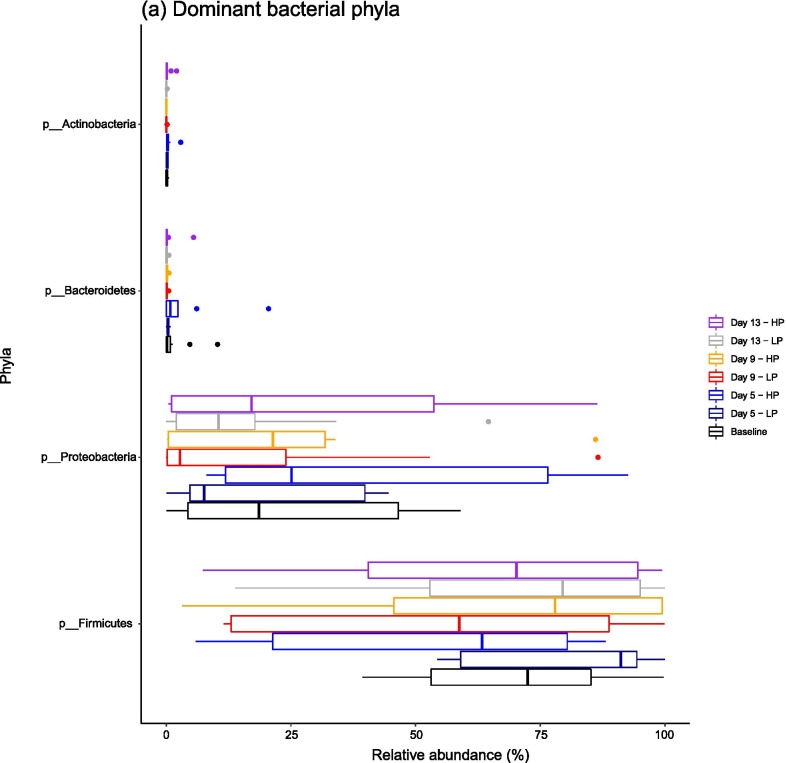

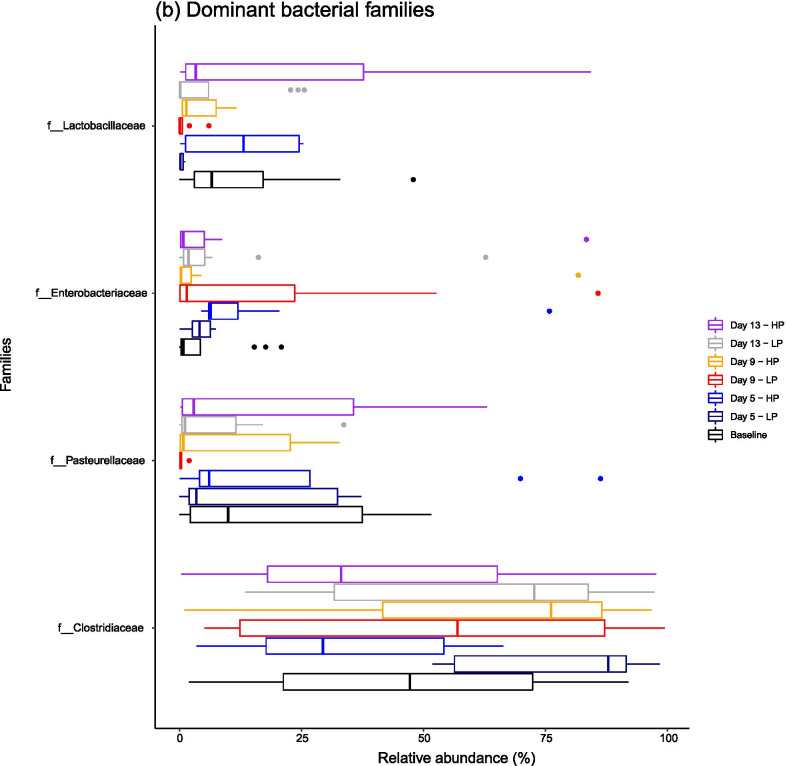
Boxplots showing the dominant **a** bacterial phyla and **b** bacterial families across all experiment groups. The ileal microbiota is highly variable between groups, but a core composition is evident

The ileal microbiota was highly variable between individuals and within treatments, but a core microbiota was evident across all samples, diets and time points. Firmicutes and Proteobacteria dominated at phylum level (Fig. 1a) with a further 19 phyla being identified by 16S rRNA gene sequencing, and 17 bacterial phyla by metagenomics, which were in low abundance. *Clostridiaceae* dominated at family level (Fig. 1b), with three other families being present in lower abundances—*Pasteurellaceae, Enterobacteriaceae and Lactobacillaceae—*with a further 99 bacterial families being rarer members of the ileal microbiota. These four families were also the most dominant in the metagenomic dataset on day 13 (Additional file 1). At genus level, 48.2% of sequences were unclassified by 16S rRNA gene sequencing, with the most dominant core classified genus being *SMB53* (mean = 14.85 ± 14.13%), followed by *Lactobacillus* (2.17 ± 3.30%). Metagenomic sequencing revealed *Lactobacillus* to be the most abundant genus on day 13 (16.26 ± 22.03%), followed by *Actinobacillus* (10.34 ± 12.17%) and *Clostridium* (7.88 ± 8.64%). A further 182 genera were identified which were present in levels lower than 1% relative abundance. Summary statistics for the metagenomic analysis on day 13 are presented in Additional file 1.

### Temporal changes in ileal microbiota alpha diversity

**Fig. 2:**
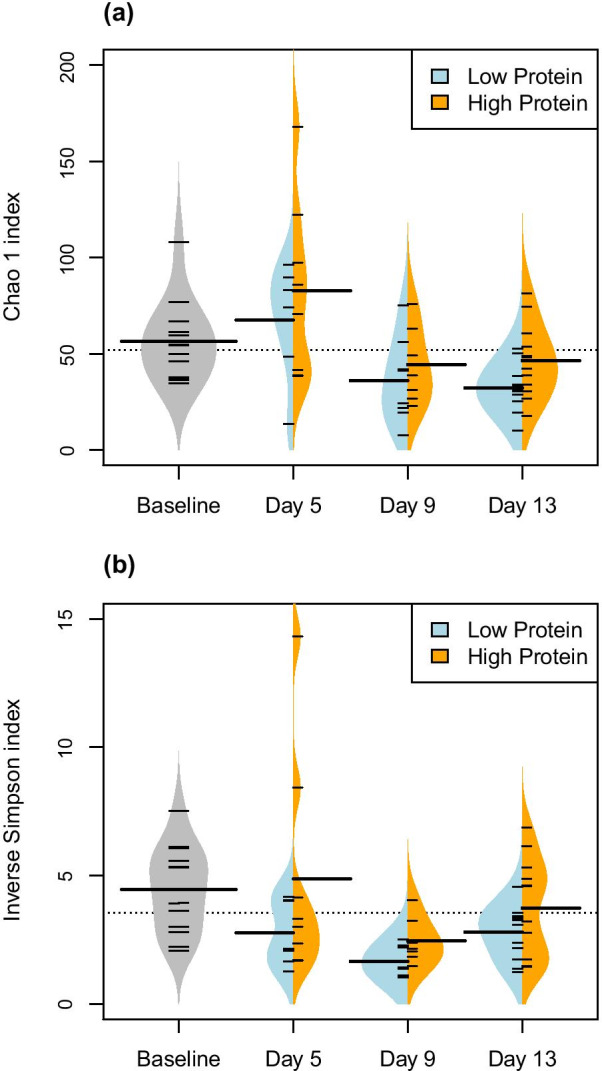
Beanplots showing the distribution of **a** Chao 1 indices and **b** Inverse Simpson indices calculated from ileal digesta samples at baseline, and at each sampling point when the pigs were fed either the LP or HP diet (Day 5–Day 13). Small black bars indicate individual data points, with the wider black lines representing the mean value

Temporal changes in ileal microbiota richness (Chao 1) and diversity (Inverse Simpson) indices are visualised in Fig. 2. Pairwise comparisons of indices between time points were carried out, exploring both main effects (i.e., time and diet) and effects within each dietary treatment. The full statistical output as calculated from the 16S rRNA gene sequencing dataset is shown in Additional file 2.

A significant reduction in microbiota richness was observed between baseline and day 13 (P = 0.02). This reduction was specific to the LP group (P < 0.001) and was not observed in the HP group independently (P = 1.00) (Fig. 2a). A significant reduction in diversity was observed from baseline to day 9 (P < 0.001) and occurred in both the LP (P < 0.001) and HP (P = 0.03) groups independently (Fig. 2b). Diversity indices then returned to levels which were not dissimilar to the baseline samples (LP: P = 0.07, HP: P = 1.00). Overall, ileal richness was not significantly different when comparing the LP and HP group (P = 0.08), but ileal diversity was significantly higher in the HP group (P = 0.04).

### Temporal changes in ileal microbiota beta diversity and taxonomy

Ileal microbiota structure changed significantly over time (P = 0.01), with changes specifically in the LP group again being observed (P < 0.01) which were not present in the HP group (P = 0.30). In the LP group, the most marked shifts in structure occurred between baseline and day 5 (main effect: P = 0.04, LP: P = 0.03) and days 5 and 9 (main effect: P = 0.06, LP: P = 0.01), with microbiota structure being stable between days 9 and 13 (main effect: P = 0.40, LP: P = 0.40).

**Fig. 3:**
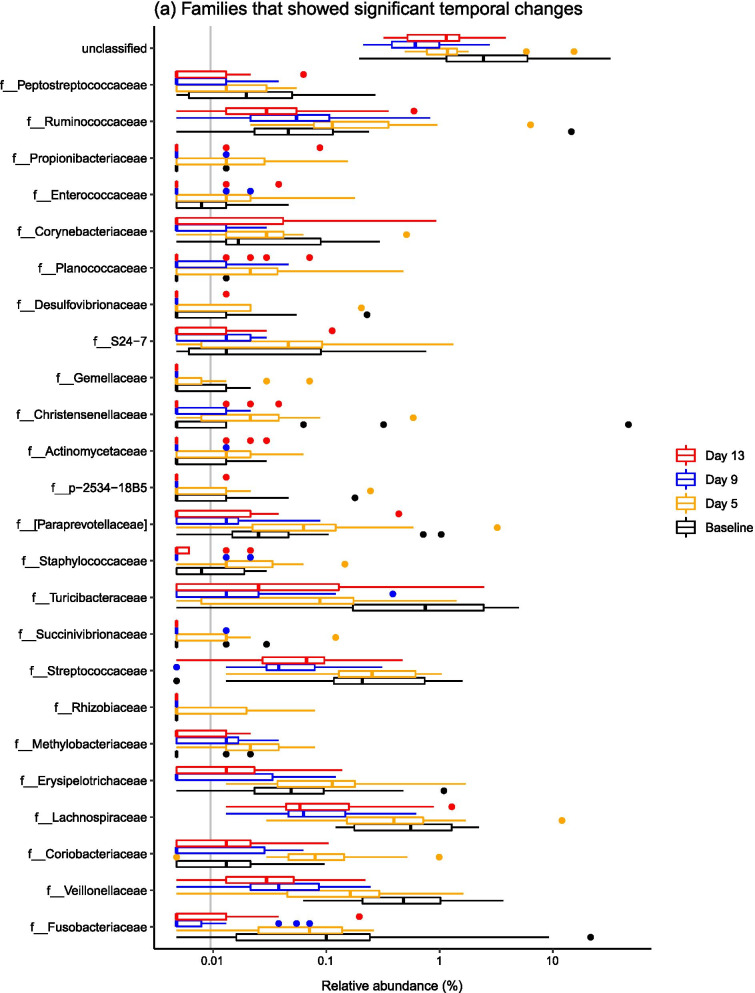

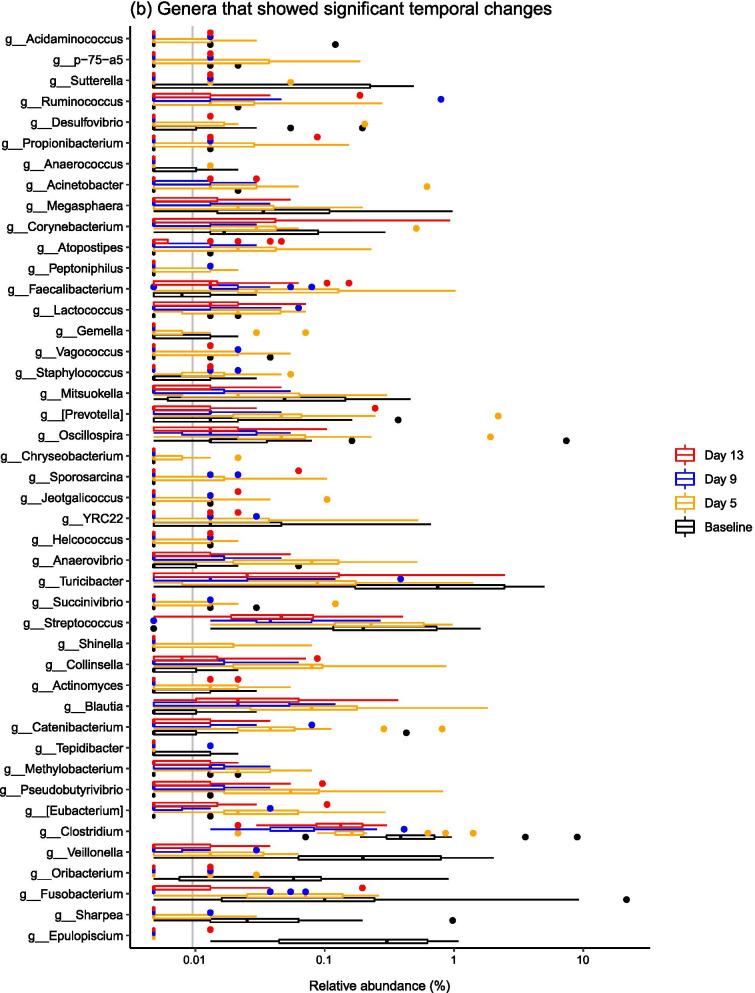
Barplots describing the bacterial taxa that changed significantly over the post-weaning period at both **a** phylum and **b** family levels across all samples, diets and time points

**Fig. 4:**
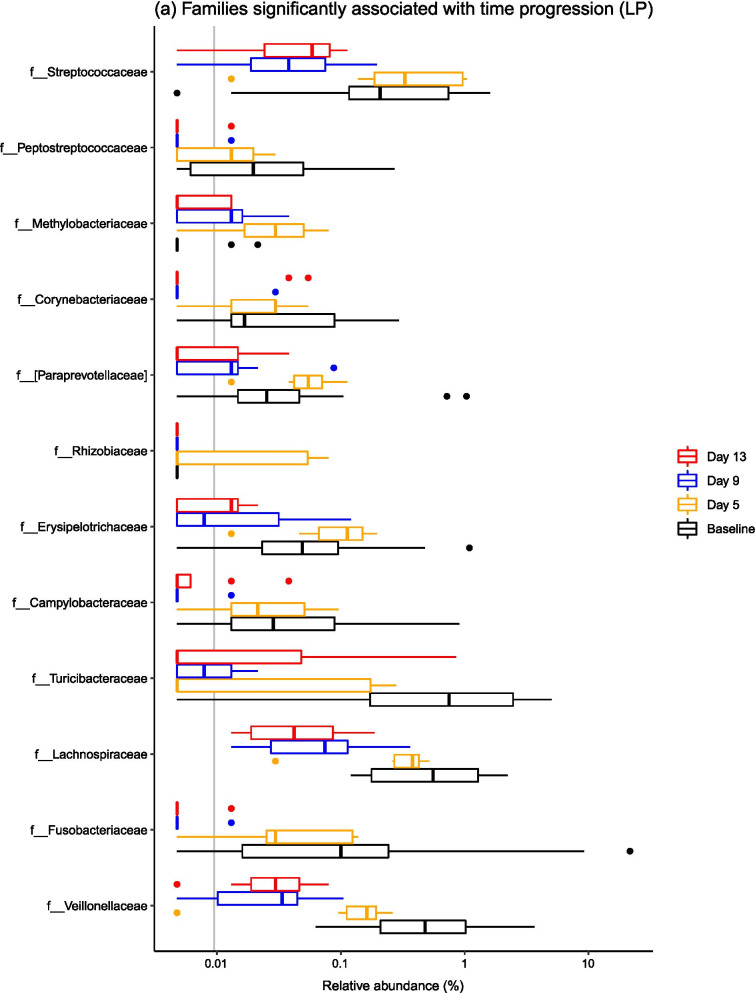

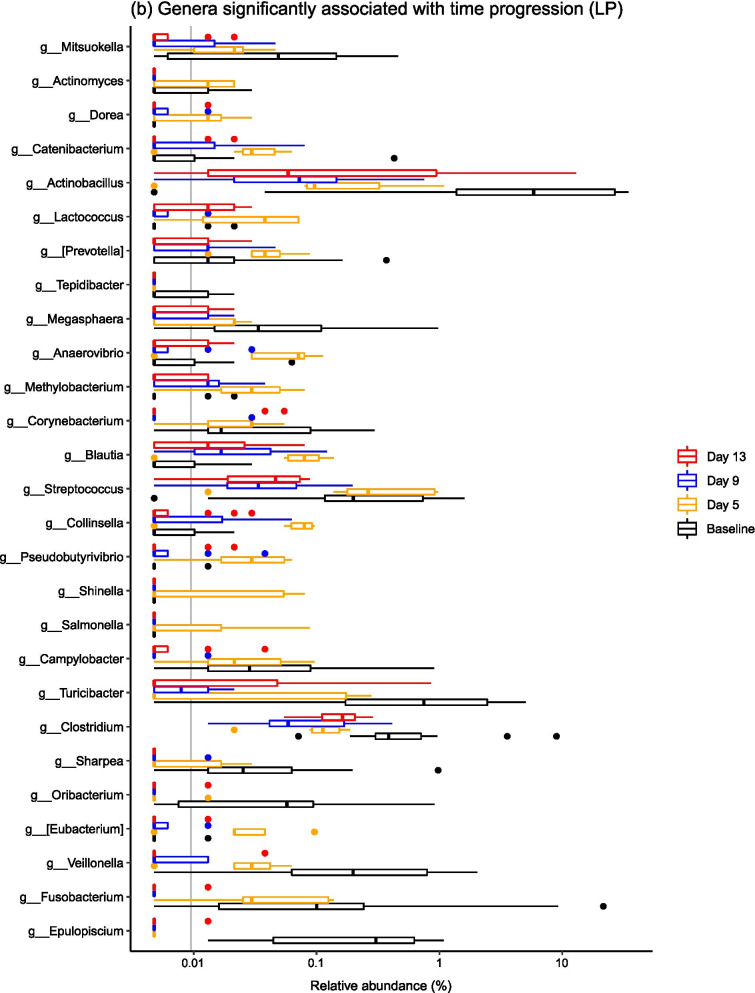

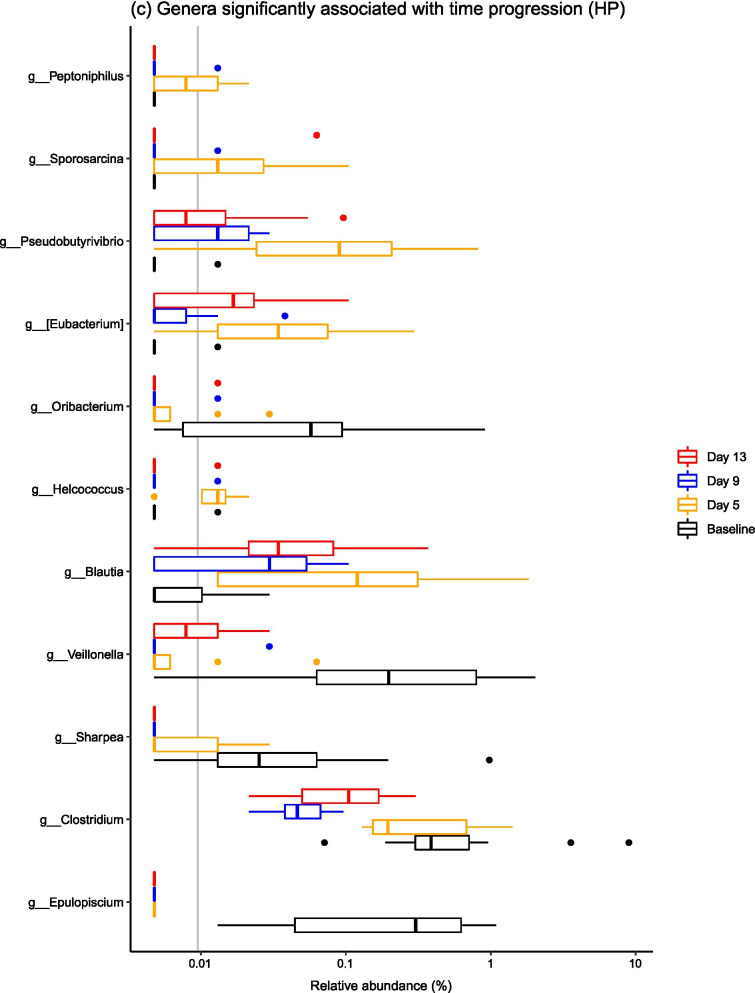
Barplots describing the bacterial taxa that changed significantly over the post-weaning period by dietary treatment—**a** shows families associated with time point in the LP group, and there were no families linked to time point in the HP group. Genera significantly associated with time progression in both the **b** LP and **c** HP groups are visualised

When considering common taxonomic changes across both dietary groups, several bacterial taxa changed significantly over time (Fig. 3). Common patterns across both groups were changes in the abundances of *Epulopiscium*, *Eubacterium*, *Oribacterium*, *Sharpea*, *Clostridium*, *Veillonella*, *Pseudobutyrivibrio* and *Blautia* (Fig. 4). Full statistical outputs are from the 16S rRNA gene sequencing dataset are presented in Additional file 3.

There were also diet-specific differences in the relative abundances of particular taxa, with most significant taxonomic changes within the LP group (Fig. 4). In the LP group, significant shifts in relative abundance occurred in 11 bacterial families and 27 bacterial genera (P < 0.05), with marked stepwise increases in *Streptococcaceae, Peptostreptococcaceae, Lachnospiraceae, Fusobacteriaceae* and *Veillonellaceae*. At genus level, stepwise increases in *Mitsuokella*, *Actinobacillus*, *Streptococcus*, *Campylobacter*, *Turicibacter* and *Veillonella* species were observed. There were less taxa associated with time progression in the HP group, with 0 bacterial families and 11 bacterial genera changing in abundance over the post-weaning period. Specifically, there were overall increases in *Pseudobutyrivibrio*, *Oribacterium*, *Veillonella*, *Clostridium* and *Epulopiscium* species.

### Diet-linked changes in the ileal microbiota

Ileal microbiota richness and structure were not significantly different when comparing the LP and HP groups at any time point (P > 0.05). When considering both dietary groups, the ileal microbiota destabilised after weaning and the transition to solid feed only (P = 0.036). Specifically, the ileal microbiota was significantly less stable in the HP group overall (P = 0.05), with the ileal microbiota being significantly less stable specifically on day 5, compared to the LP group (P = 0.008).

On day 5, both *SMB53* (LP: 18.75 ± 1.63%, HP: 9.31 ± 1.89%, P = 0.0002) and an unclassified *Clostridiaceae* (LP: 56.59 ± 8.50%, HP: 24.78 ± 6.63%, P = 0.002) were in significantly greater abundance in the LP group. In contrast, *Lactobacillus reuteri* was in significantly greater abundance in the HP group than in the LP group (LP: 0.37 ± 0.00%, HP: 10.97 ± 4.04%, P = 0.004). On day 9, *SMB53* then became more significantly more dominant in the HP group (LP: 6.35 ± 2.37%, HP: 27.50 ± 10.24%, P = 0.03), along with an unclassified *Pasteurellaceae* (LP: 0.23 ± 0.00%, HP: 6.04 ± 2.87%, P = 0.03) and *Haemophilus parasuis* (LP: 0.00 ± 0.00%, HP: 2.66 ± 1.31%, P = 0.03). On day 13, there were no significant differences in relative abundances when comparing both dietary groups.

The mean relative abundance of *Actinobacillus porcinus* was markedly higher in the HP group on day 5 (LP = 0.32 ± 0.00%, HP = 2.12 ± 2.90%), day 9 (LP = 0.17 ± 0.26%, HP = 2.51 ± 3.23%) and day 13 (LP = 1.97 ± 3.91%, HP = 9.79 ± 13.40%), which defined the HP group (P = 0.04).

### Metagenomic and microbiota-weight gain interaction data

Average daily weight gain data ranged from 11.73 to 62.57 g/day/kg weaning weight (Additional file 5). There were no statistically significant correlations observed between ADWG and the relative abundances of particular OTUs (P > 0.05). Two enterotypes were assigned to the taxonomic profiles generated from the porcine ileal samples, but this partitioning was not linked to ADWG measurements (Additional file 5).

Metagenomic data was generated using samples from day 13 only to allow deeper taxonomic analysis and comparisons between the low and high protein groups later into the crucial post-weaning period. At kingdom level, the ileal microbiota was comprised of Eukaryota (29.06 ± 14.29%), Bacteria (70.50 ± 14.39%), Viruses (0.16 ± 0.00%) and Archaea (0.20 ± 0.00%). Members of the Eukaryota were predominantly host DNA contaminants, and so were not considered for analysis. Additionally, due to the relatively low levels of Viruses and Archaea in this dataset, only bacterial sequences are described. All taxonomic groups identified in the metagenomic dataset at kingdom, phylum, family and genus level are described in full in Additional file 1. To summarise, at phylum and kingdom levels, there were no significant differences between groups (P > 0.05). At family level, *Neisseriaceae* were present at significantly higher levels in the HP group (P = 0.03).

Table 1 describes the bacterial genera which were present in significantly different relative abundances at the final sampling point. All statistical analyses carried out at phylum, family and genus level are described in full in Additional file 4.

**Table 1:** Bacterial genera present in significantly different relative abundances when comparing the low and high protein diet groups

Taxa	Mean (LP)	Mean (HP)	SD (LP)	SD (HP)	Adjusted P value
*Actinobacillus*	2.785	16.249	3.583	13.712	0.032
*Agarivorans*	0.001	0.004	0.002	0.004	0.047
*Aggregatibacter*	0.061	0.574	0.087	0.701	0.026
*Avibacterium*	0.047	0.419	0.074	0.535	0.030
*Basfia*	0.024	0.305	0.032	0.405	0.027
*Bibersteinia*	0.021	0.150	0.030	0.161	0.047
*Catenovulum*	0.001	0.010	0.002	0.013	0.018
*Gardnerella*	0.000	0.004	0.000	0.008	0.033
*Glaesserella*	0.480	3.098	0.643	2.604	0.030
*Haemophilus*	0.199	1.571	0.268	1.845	0.018
*Histophilus*	0.024	0.197	0.033	0.219	0.018
*Kingella*	0.000	0.008	0.000	0.007	0.018
*Mannheimia*	0.121	0.683	0.140	0.651	0.020
*Neisseria*	0.012	0.086	0.015	0.073	0.018
*Pasteurella*	0.094	0.647	0.145	0.714	0.018
*Rodentibacter*	0.020	0.151	0.029	0.178	0.03
*Wolinella*	0.000	0.002	0.000	0.002	0.024

Many of the organisms that are present in significantly different relative abundances are members of the *Pasteurellaceae* family, and the genera significantly different in Table 1 represent a mean relative abundance of 3.9% and 24.1% of the ileal microbiota in the LP and HP groups, respectively. As reflected in the 16S rRNA gene sequencing data, *Actinobacillus* species were present in a higher relative abundance in the HP group, and so are an indicator of and a dominant ileal microbiota member in the HP group.

Carbohydrate active enzymes (CAZymes) were identified in this dataset, which are responsible for the synthesis and breakdown of carbohydrates and are key determinants for both composition and activity of the gut microbiome [10]. The abundances of these were compared between high and low protein samples, excluding a sample from the LP group due to this sample having few CAZyme genes. 142 families of CAZymes were identified: 5 auxiliary activities families, 17 carbohydrate-binding module class families, 12 carbohydrate esterase families, 62 glycoside hydrolase families, 41 glycosyltransferase families, 4 polysaccharide lyase families and cohesins. HP and LP samples did not cluster significantly separately by their CAZyme composition. However, GH33 exosialidases were found to be significantly more abundant in the HP group (adjusted P value = 0.0063).

## Discussion

In this study, we examined the bacterial composition of ileal digesta samples taken before weaning. Piglets were then assigned to one of two diets—low protein or high protein—and further samples studied to establish how the ileal microbiota developed during the weaning transition period. The main limitation of this study is that the same individuals could not be sampled repeatedly using this experimental design. However, this methodology allowed us to identify core temporal changes in the ileal microbiota, and dietary associations with particular taxa and microbiota function. There have been many lines of research and practices aiming to limit the impact of this challenging growth period, yet there is no baseline description of microbiota dynamics during the weaning transition.

### The challenges of studying ileal bacterial communities

There are fewer studies on the small intestinal microbiota in comparison to faecal samples, and as such, the challenges associated with studying these communities are not as well known. Firstly, the variability between individuals makes it more difficult to explore core key changes over time and/or between treatments, meaning that a greater number of samples are often required. Secondly, when utilising metagenomics to study lower biomass intestinal environments, the relatively high proportion of host DNA contamination can be problematic. A high level of eukaryotic DNA was discovered in this study (which was predominantly of suspected porcine origin), which had an impact on sequencing coverage and was consequently removed from the analysis. Plant-associated DNA was also detected in all samples, with feed components being the source, but these sequences were not removed as we present a descriptive taxonomic analysis, rather than a structural analysis. Thirdly, at genus level, 48% of the 16S rRNA gene sequences remained unclassified, which is in part due to the limitations of using a short fragment of the 16S rRNA gene for sequencing and public sequence databases not yet encompassing a large range of bacterial sequences required for classification [11]. Consequently, we advise that greater sample numbers should be considered for future studies and that higher sequencing coverage may be required to make comparisons between groups.

### The ileal microbiota was destabilised by introduction to feed

Ileal microbiota richness decreased significantly over the post-weaning period but was highly variable at an individual level. Ileal microbiota richness decreased significantly in pigs fed the low protein diet but not the high protein diet. Other authors have previously reported that porcine ileal microbiota richness decreases with lower dietary protein intake [12–14], perhaps indicating that a lower protein diet suppresses the growth of protein-fermenting bacteria [1]. Additionally, significant reductions in microbiota diversity occurred in both dietary groups between baseline and day 9, suggesting that the sudden introduction to a solid diet caused significant shifts in microbiota composition, with particular bacterial groups increasing in dominance (as discussed below). Other authors have reported that the most pertinent driver of microbiota shifts is the transition period between nursing and introduction to solid feed in all gut sections, not just the small intestine [15], supporting the view that nutrition is an important influence in the development of the porcine gut microbiota.

### The ileal microbiota was dominated by few taxa

We found that the ileal microbiota was low in diversity, compared to porcine faecal samples from pigs of the same age in our previous work [1, 16], and was heavily dominated by Firmicutes and Proteobacteria. This has been observed in many other studies on the porcine ileal microbiota in pigs of comparable age [7, 17] and in older pigs [18–21]. This clear dominance at phylum level in older pigs was driven by the high abundance of *Anaerobacter* and *Turicibacter* at 90 days old [21], *Lactobacillus* at 150 days old [19] and *Clostridium* at 300 days old [20]. In piglets of comparable age to our study, it was found that the ileal and mucosal microbiota were dominated by *Lactobacillus*, *Clostridiaceae* and *Turicibacter* [7] and *Peptostreptoccaceae* and *Streptococcaceae* [18]. Here, *Clostridiaceae* comprised a median relative abundance of 53%, which by far exceeded the relative abundances of the other core families (i.e. *Pasteurellaceae, Enterobacteriaceae and Lactobacillaceae*, which made up between 1 and 3% of ileal communities). The dominance of *Clostridiaceae* in the porcine ileum has been reported elsewhere, with members of this bacterial family having the ability to metabolise plant-derived saccharides such as glucose derived from bacterial breakdown of starch or non-starch polysaccharides [2].

Within these dominant phyla and families, there were also few dominant bacterial genera. *SMB53* was highly enriched in the small intestine in our study, which has also been the case in other work [20, 22]. The role of *SMB53* is not well known [23] and has been found to be most closely related to the genus *Clostridium* [24]. We also found at genus level, the dominant taxa were *Clostridium*, *Lactobacillus*, *Actinobacillus* and *Mycoplasma*. These and other taxa residing in the ileum have the ability to thrive in the harsh conditions of this gut section, with many being linked to feed efficiency and nutrient utilisation [25, 26]. Some taxa isolated here, such as *Actinobacillus* and *Mycoplasma* species, are opportunistic pathogens.

### The ileal microbiota composition changed significantly over time

Microbiota composition was highly variable between pigs, but it is well recognised that the ileal microbiota is less stable compared to the faecal microbiota [1, 18]. It is thought that the higher variability in the midgut is due to the lower bacterial diversity and biomass in comparison to the hindgut, and the constant influx of bacteria from food and the environment that will variably survive the relatively low pH in the stomach, as well as the shorter digesta transit time [2, 27]. Additionally, the structure of the gut, chemical and nutrient gradients and presence of antimicrobial peptides are also thought to contribute to spatial differences in the gut microbiota [27]. In spite of this variation, significant temporal changes in the ileal microbiota were observed. Ileal microbiota structure changed significantly over a short time period post-weaning, which was underpinned by changes in the relative abundances of several bacterial taxa. Specifically, temporal changes in the abundances of *Epulopiscium*, *Eubacterium*, *Oribacterium*, *Sharpea*, *Clostridium*, *Veillonella*, *Pseudobutyrivibrio* and *Blautia* occurred in both dietary groups. *Epulopiscium* was present in the pre-weaned porcine gut only, and was not detected at any further time points, and has been found to be a dominant member of the sow-fed neonatal pig ileum [28], with *Oribacterium* and *Sharpea* also diminishing post-weaning. The dominance of *Clostridium* species and the other Clostridia mentioned (i.e. *Eubacterium*, *Oribacterium*, *Veillonella*, *Pseudobutyrivibrio* and *Blautia* species) in the post-weaned pig ileum has been described elsewhere [2, 29]. Commensal Clostridia such as these have been shown to have an important role in gut homeostasis [30], which may contribute to the greater stability of the porcine ileal microbiota during later growth phases.

### There were no links between microbiota composition and weight gain

In our previous study [1], we established that average daily weight gain was not significantly different between the LP and HP pigs, but the ileal microbiota was significantly different when comparing the two dietary groups. Here, we sought to establish whether average daily weight gain was linked to these differences in microbiota composition. We found that microbiota structure and specific OTU relative abundances were not significantly linked to weight gain. In previous studies, correlations between the small intestinal microbiota composition and weight gain have been evident in both pre-weaned pigs [31] and in finisher pigs [32]. We have shown that the ileal microbiota is highly destabilised during the weaning transition period, and hypothesise that the ileal microbiota is consequently not optimal at this stage to utilise the available nutrients in solid feed to improve host growth performance.

### There were diet-specific differences in the ileal microbiota

There were several bacterial taxa in significantly different abundances when comparing the two dietary groups. As with the richness and diversity indices, these taxonomic changes were more marked in the low protein group. *Lactobacillus reuteri* was present in higher abundances in the high protein group. Some members of the *Lactobacillus* genus have proteolytic properties [33] and may have increased in abundance due to greater protein availability [1]. Higher *Lactobacillus* species abundance has also been linked to increased feed efficiency [22]. Specifically, *Lactobacillus reuteri* has been used as a probiotic in pigs and has demonstrated strong adhesion to the porcine gut, in addition to roles in competitive exclusion and improved host performance [34].

In addition*, Haemophilus parasuis* and several members of the *Pasteurellaceae* were in higher abundance in the high protein group. These organisms are key pathogens in pigs, and the marked difference between the two groups is of potential biological significance. Although many members of the *Pasteurellaceae* are commensal gut constituents, the gut acts as an important reservoir of porcine pathogens that can cause serious systemic diseases [35]. High protein diets have been shown to have a higher acid-binding capacity and can therefore lead to an increased gut pH [36], providing a more favourable environment for pathogenic bacteria [37].

Another member of the *Pasteurellaceae*, *Actinobacillus* species, was a clear indicator of the HP group, which was revealed in both the 16S rRNA gene and metagenomic data sets. Specifically, *Actinobacillus porcinus* was in a markedly greater relative abundance in the HP group throughout the experiment. Other work has revealed that *Actinobacillus porcinus* was in greater abundance in low residual feed intake pigs [25] and *Actinobacillus* species were enriched in low fatness pigs [20], and so this genus may be a key performance indicator in pigs.

Finally, using metagenomic sequencing, we identified CAZyme genes and compared the abundance of these between dietary groups. These were targeted due to their role as key determinants for both the composition and activity of the microbiome in weaner pigs [10] and their essential role during the transition between milk and solid feed [38]. The dietary groups did not cluster significantly separately by their CAZyme composition, though the Glycoside Hydrolase Family 33 (GH33) was more abundant in the HP group. GH33 is a family of exosialidases, which catalyse the removal of sialic acid residues to release free sialic acid. Free sialic acid is used as a carbon and energy source for bacteria. Sialidases are known to affect host-microbe interactions on the gut mucosa [39, 40] and have been suggested to play a role in the pathogenicity of particular micro-organisms [41]. Hosting a microbiota with the ability to produce and/or consume sialic acid in the gut has been said to be essential for gut homeostasis [39]. The therapeutic potential of the modulation of sialidase expression has been raised, through the use of specific inhibitors or pre- and probiotics to target specific members of the microbiota [39].

## Conclusions

The ileal microbiota changed rapidly over the post weaning period and was de-stabilised by the introduction to a solid diet. Ileal microbial communities were highly variable between individuals, with no observed links with weight gain, but core changes in specific bacterial taxa were observed. Pigs fed the high protein diet had less stable ileal microbiomes, higher abundances of key porcine pathogens and higher levels of GH33 exosialidases—which can influence host-microbe interactions and pathogenicity. Targeted microbiome studies improve our understanding of the complexity and importance of gut microbiota dynamics and may lead to the development of alternative management strategies to improve host health and performance during the weaning transition period.

## Methods

### Pigs, housing and dietary treatments

Pigs (Large White × Landrace) were weaned (day 0) at 25.0 ± 0.8 days of age (mean ± SD) and weighed 9.11 ± 1.42 kg. The pigs were housed in 4 m^2^ square pens in groups of four with a single feeder and nipple drinker, and the pen flooring was cleared and bedded with fresh sawdust daily. Pens were balanced for weaning weight, sex and litter origin within each of the four experimental rounds (Additional file 5) with the full experimental design being described previously [1].

Before weaning, the piglets were administered a standard creep feed (digestible energy = MJ/kg, CP = 230 g/kg) from day − 7 to day 0 (i.e., weaning). Ileal samples (n = 72) were collected from euthanised pigs before weaning (n = 8) and the remaining pigs (n = 64) were assigned to the low protein (LP) (n = 32) or high protein (HP) (n = 32) diet. Detailed compositional data describing these experimental diets have been published previously [1], with a crude protein level of 180.9 g/kg and 228.8 g/kg being measured from the formulated LP and HP diets, respectively. Pigs were fed ad libitum for the trial duration.

As these pigs were recruited as controls for an infection study, whilst housed separately from their challenged counterparts, a sham inoculation with phosphate buffered saline (PBS) was carried out on day 2 of the experiment.

### Ileal digesta sampling

Temporal ileal digesta sampling was carried out, whereby pigs were subject to post-mortem on days − 1 (i.e. the day before weaning and prior to treatment assignment), 5, 9 and 13. At post-mortem points from day 5–13, pigs were selected from each pen to maintain balance across dietary treatment for weaning weight, sex and litter origin.

Pigs subject to post-mortem were first sedated (medetomidine—0.01 ml/kg at 1 mg/ml, midazolam—0.1 ml/kg at 5 mg/ml, ketamine—0.1 ml/kg at 100 mg/ml and azaperone—0.025 ml/kg at 40 mg/ml) and then euthanised by intracardiac injection of pentobarbital (0.7 ml/kg at 200 mg/ml). The abdomen was dissected from pubis to sternum to allow clear visualisation of the gastrointestinal tract. The caecum was identified and tied off at the ileal-caecal junction using string, and a 10 cm section of ileum was measured out before tying off again with string. The tied-off ileal section was then cut out and removed, before emptying the ileal contents into a DNase- and RNase-free universal tube and immediately placing on dry ice prior to DNA extraction.

### 16S rRNA gene sequencing

All ileal digesta samples were subject to DNA extraction and 16S rRNA gene sequencing targeting the V3 hypervariable region, using the principles [11] and the methodology as described previously [16]. Further details regarding DNA quality control, the use of sequencing controls and sequencing details for this study are described in published work [1]. Briefly, DNA extractions were carried out using the MoBio PowerSoil DNA isolation kit as per the manufacturer’s instructions (now branded as the DNeasy PowerSoil kit; Qiagen, United Kingdom) using 500 mg of ileal contents. The yield and quality of the DNA extracts were assessed using a NanoDrop spectrophotometer (Thermo Scientific, United Kingdom) and by running extracts on a 2% agarose gel. The V3 hypervariable region of the 16S rRNA gene was amplified using dual-index primers 341F (5′-CCTACGGGAGGCAGCAG-3′) and 518R (5′-ATTACCGCGGCTGCTGG-3′) as previously described [1] and submitted for sequencing (Edinburgh Genomics, United Kingdom) using the Illumina MiSeq platform (Illumina, United States) and V2 chemistry, producing 250 bp paired-end reads. The generated sequences from ileal contents for this study were processed using other [42] (version 1.36.0) as detailed previously [1] using the Greengenes database trimmed to the V3 hypervariable region to improve classification depth. To enable focus on temporal shifts in the ileal microbiota, a sub-set of data from our previous study [1] was re-analysed to gain better resolution for both descriptive and statistical analyses.

A total of 130,123 ± 67,133 contiguous sequences were generated, with 91,143 ± 57,015 remaining after quality control. The remaining sequences were clustered into 681 operational taxonomic units (OTUs) using a database-dependent method. 5.4% of all sequences were not classified further than “Bacteria”. Sequence data were sub-sampled for analysis (n = 10,000) to minimise the effects of uneven sampling for statistical analysis. A Good’s coverage value of 0.99 was calculated for each of the samples.

The Chao 1 index and Inverse Simpson Index (ISI) were calculated to study richness and alpha diversity, respectively. To test statistical significance of differences in alpha diversity between groups, the Pairwise Wilcoxon Rank Sum Test (with Bonferroni corrections for multiple comparisons) was used. A distance matrix was built using Yue and Clayton theta similarity coefficients [43], which inputs both community membership and relative abundance data. Any statistically significant clustering by group was assessed using analysis of molecular variance (AMOVA) [44]. The statistical significance of variation between populations was assessed using homogeneity of molecular variance (HOMOVA) [45]. Metastats [46] was used to identify taxa that were in significantly different abundances when comparing dietary groups, and the P values were corrected using the false discovery rate. An indicator analysis was run to identify taxa which were significantly more abundant in specific treatment groups [47]. In order to identify bacterial phyla and families that were present in significantly different relative abundances between groups, Kruskal–Wallis tests were run with Bonferroni corrections being applied. Both statistical analyses and graphs were generated using R (version 3.5.1).

### Shotgun metagenomic sequencing

A subset of ileal DNA extracts obtained on day 13 (n = 14) were submitted to the sequencing centre (Edinburgh Genomics, United Kingdom). Illumina TruSeq DNA Nano libraries were prepared, and sequencing was carried out using the HiSeq 4000 platform generating 150 bp paired-end reads (Illumina, United States). Two samples from the LP group failed sequencing (due to low coverage of microbial DNA), so 12 samples were taken forward for analysis.

Illumina adaptors were removed using trimmomatic (v.0.36) [48]. Reads were mapped to the *Sus scrofa* reference genome using BWA MEM (v.0.7.15) [49] and only reads that did not map to this reference genome were taken forward for further analysis. A custom Kraken2 database was constructed consisting of RefSeq complete genomes and the *Sus Scrofa* reference genome; taxonomies were then assigned to paired end reads using Kraken2 (v.2.0.8-beta) [50], whereby 46.87 ± 13.95% of reads were unclassified to this database.

Assemblies were performed using IDBA-UD (v.1.1.3) [51] with the options –num_threads 16 –pre_correction –min_contig 300. Protein coding sequences were identified in assemblies using Prodigal (v. 2.6.3) [52]. To identify carbohydrate-active enzymes (CAZymes), proteins were compared to the CAZy database using dbCAN2 (version 7, 24^th^ August 2018) [53]. CAZyme abundances were calculated as the sum of the reads mapping to assembly proteins after using DIAMOND (v0.9.21) [54] to align reads to proteins. Data were subsampled to the lowest sample abundance prior to statistical analyses (excluding Deseq analysis). R (version 3.5.1) was used for all statistical analyses. Comparisons between groups were performed using the donis function (PERMANOVA) from the vegan package. Deseq2 was used to calculate differences in abundance of individual taxonomies and CAZymes [55].

### Average daily weight gain and microbiota composition

All pigs were weighed at days − 1 (baseline pigs only), 0, 2, 5, 9 and 13 post-weaning. The data from pigs sampled on day 13 (Additional file 5) was used to calculate the average daily weight gain (ADWG) expressed as grams per day normalised using the weaning day weight. These data were then used to test whether ADWG was linked to microbiome composition. Firstly, a Dirichlet Multinomial Mixtures (DMM) model [56] was run to assess whether higher ADWG values were linked to a specific enterotype. Secondly, a Pearson correlation was run using an axes file generated using the AMOVA output file to assess whether ADWG was linked to changes in relative abundances of specific OTUs.

## Supplementary Information

**Additional file 1.**  Taxonomic description of metagenomic data (day 13).

**Additional file 2.** Alpha diversity statistical outputs from 16S rRNA gene sequencing data.

**Additional file 3.** Statistical analysis of taxonomic changes by time point.

**Additional file 4.** Full statistical analysis of metagenomic dataset highlighting taxonomic differences between diets at day 13.

**Additional file 5.** Pig metadata.

## Data Availability

The generated raw 16S rRNA gene sequence fastq files (with primers removed) and raw metagenomic paired-read fastq files are available publicly through the European Nucleotide Archive (ENA) under accession number PRJEB33396. All data analysed during this study are described in the manuscript, with statistical outputs and sample metadata being made available in the supplementary information files and within the ENA submission.

## Abbreviations

16S rRNA: 16S ribosomal ribonucleic acid
ADWG: Average daily weight gain
AMOVA: Analysis of molecular variance
CAZyme: Carbohydrate-active enzymes
CP: Crude protein
GH33: Glycoside hydrolase family
HOMOVA: Homogeneity of molecular variance
HP: High protein
ISI: Inverse Simpson index
LP: Low protein
OTU: Operational taxonomic unit
